# Scaling up from greenhouse resistance to fitness in the field for a host of an emerging forest disease

**DOI:** 10.1111/eva.12080

**Published:** 2013-07-19

**Authors:** Katherine J Hayden, Matteo Garbelotto, Richard Dodd, Jessica W Wright

**Affiliations:** 1 Environmental Science Policy, and Management, University of CaliforniaBerkeley, CA, USA; 2 Pacific Southwest Research Station, USDA Forest ServiceDavis, CA, USA

**Keywords:** forest management, generalized linear mixed models, host–parasite interactions, invasive species, natural selection and contemporary evolution, quantitative genetics, sudden oak death, survival analysis

## Abstract

Forest systems are increasingly threatened by emergent, exotic diseases, yet management strategies for forest trees may be hindered by long generation times and scant background knowledge. We tested whether nursery disease resistance and growth traits have predictive value for the conservation of *Notholithocarpus densiflorus*, the host most susceptible to sudden oak death. We established three experimental populations to assess nursery growth and resistance to *Phytophthora ramorum*, and correlations between nursery-derived breeding values with seedling survival in a field disease trial. Estimates of nursery traits’ heritability were low to moderate, with lowest estimates for resistance traits. Within the field trial, survival likelihood was increased in larger seedlings and decreased with the development of disease symptoms. The seed-parent family wide likelihood of survival was likewise correlated with family predictors for size and resistance to disease in 2nd year laboratory assays, though not resistance in 1st year leaf assays. We identified traits and seedling families with increased survivorship in planted tanoaks, and a framework to further identify seed parents favored for restoration. The additive genetic variation and seedling disease dynamics we describe hold promise to refine current disease models and expand the understanding of evolutionary dynamics of emergent infectious diseases in highly susceptible hosts.

## Introduction

The global trade in plants and plant products has inevitably led to dissemination of plants’ microscopic associates and has been repeatedly accompanied by devastating disease outbreaks in natural systems (Anderson et al. 2004; Desprez-Loustau et al. 2007; Brasier 2008; Loo 2009). Dramatic losses of species due to introduced forest pathogens have been documented for at least 100 years, among others, as the loss of overstory *Castanea dentata* to chestnut blight (Anagnostakis 1987); and new diseases continue to emerge, including redbay wilt in the southeast United States (Fraedrich et al. 2008), and ash dieback in Europe (Kowalski 2006). These introductions pose a global experiment in species interactions as well as an opportunity and a challenge for management and evolutionary theory to inform each other. The value of an evolutionary perspective for the management of systems set out of equilibrium by species introductions and changing climates is becoming increasingly clear (Antia et al. 2003; Stockwell et al. 2003; Schoettle and Sniezko 2007; Lankau et al. 2011); conversely, the outcome of evolution-informed management strategies present an opportunity to test predictions made from models and simpler systems.

California forests are now in the early stages of an epidemic caused by the introduced pathogen *Phytophthora ramorum*. First observed in the mid-1990s on dying *Quercus* and tanoak (*Notholithocarpus densiflorus*) trees in two northern California counties, *P. ramorum* has been found since to infect nearly every plant species in the coastal mixed-evergreen understory across hundreds of kilometers (Rizzo and Garbelotto 2003; Garbelotto and Hayden 2012). In wildlands, *P. ramorum* populations consist of relatively few clonally reproducing lines descended from only one of four major lineages known worldwide; evidence suggests that these wild populations have escaped from nurseries (Mascheretti et al. 2008).

Tanoaks are thus far the hosts most susceptible to the fatal disease form, sudden oak death (SOD), having been killed in the hundreds of thousands (Meentemeyer et al. 2008), with up to 100% plot-level mortality (Davis et al. 2010). Tanoaks are endemic to the California Floristic Province (Hickman 1993), and while *P. ramorum* is established in only a subset of that range, the infested area expands yearly (Geospatial Innovation Facility and Kelly Research and Outreach Lab 2011). Despite their importance in Native American cultures (Ortiz 2006; Bowcutt 2011), and their ecological importance as the primary nut producer and ectomycorrhizal host in some forest systems (Bergemann and Garbelotto 2006), they received little academic study prior to the SOD epidemic, except as an impediment to conifer growth (Bowcutt 2011). Until recently (Nettel et al. 2009; Hayden et al. 2011), little was known about tanoak phylogeography, breeding system, or population structure.

The evolution-based management of long-lived species for which there is a paucity of background information faces an inherent problem: decisions must frequently be made from relatively short-term studies undertaken in the lab or nursery, far from the true environmental and selective forces. Additive genetic variance and its relationship with phenotype are of central importance for natural or breeder-assisted selection, but they are dependent on environment (Fisher 1918; Wright 1921), and the relationship between additive genetic variance in the laboratory and in the wild has long been a question in conservation and quantitative genetics (Gupta and Lewontin 1982; Riska et al. 1989; Conner et al. 2003). The problem is especially difficult when measurement of the trait of interest is possible only in an artificial environment, as for some disease resistance traits. In some cases, such as *Eucalyptus marginata* and *P. cinnamomi*, plants may be artificially inoculated in the field (Stukely et al. 2007), or, as in *Fraxinus excelsior* and *Hymenoscyphus pseudoalbidus*, natural infection rates may be sufficiently uniform with visible disease progression so as to allow assessment of resistance directly in transplanted seedlings within a few years (Kjaer et al. 2012). In others, however, the risk of spread and quarantine regulations may preclude artificial inoculation of plants in the wild, while natural infection events may be impossible to observe, and/or so stochastically occurring as to make waiting for natural infection impractical or impossible. An observable character, such as reduced disease response after inoculation in the nursery or lab, must therefore serve as a proxy for the desired outcome: the increased survival of a genotype or progeny line in some field environment (Sniezko 2006).

The sudden oak death–tanoak interaction is one such system in which purely field-based study is impracticable: despite its heavy ecological impacts, *P. ramorum* spread is stochastic, occurring primarily by rain splash (Davidson et al. 2005; Hansen et al. 2008; Mascheretti et al. 2008), with an estimated 2.5-year median time to infection for small tanoak stems in infested areas (Cobb et al. 2012), and asymptomatic infections have been reported (Hansen et al. 2005). For these reasons, we established a common garden of open-pollinated seed to determine the extent and distribution of additive genetic variation in growth traits in a nursery population (experiment N) and used laboratory inoculation assays to better characterize resistance to *P. ramorum* in tanoak (experiments R1, R2). To determine how traits measured in a nursery setting interact with each other and may predict fitness under natural infection dynamics, a concurrent field trial in an infested forest site was established (experiment F). While epidemiological and ecological constraints preclude the sole use of field studies in this system, we present it here as a point of comparison to nursery experiments.

## Materials and methods

### Establishment of a common garden

Open-pollinated seeds were collected in August—October 2006 from *N. densiflorus* trees (minimum 20 m apart) in five source populations, chosen to survey maximum variation (Table 1). Seeds were stored in sealed plastic bags at 4–6°C and in a 5-day period in December 2006 were planted into 6.4 cm × 25 cm conical pots under 50% shade in Berkeley, CA. Pots were covered with screen to exclude predators until germination. Each tree's seeds were divided into three groups: nursery growth studies, N; inoculation resistance studies R1, R2; and field resistance studies, F. Growth and resistance studies groups were maintained in a common location by different lab groups in this collaborative effort. Acorns designated for the study N were potted in Pro-Mix potting medium (Premier Tech Horticulture, Rivière-du-Loup, QC, Canada) and repotted into 4 cm ×4 cm × 36 cm pots at 1-year post-planting. Acorns designated for R and F studies were potted in UC mix (Baker 1957); R seedlings were not repotted prior to the studies described here; and F seedlings were transplanted into the field site at 12 months after sowing.

**Table 1:** Sites of collection of *Notholithocarpus densiflorus*, in California unless otherwise noted, with seeds collected directly from parent trees between August and October 2006 and planted in common garden in Berkeley, CA (37°52′N, 122°16′W)

Collection site*	Genetic zone†	Abbr.	Lat., Long.	Germ. ‡	N§	R1§	R2§	F§
O'Brien^RD^ (Josephine Co., OR)	Klamath	OB	42°00′N, 123°43′W	41%	24	21	3	11
Mendocino^RD^ (Mendocino Co.)	North coast California	MD	39°25′N, 123°47′W	16%	5	3	0	0
Blodgett Forest Research Station^RD^ (El Dorado Co.)	Sierra Nevada	BL	38°55′N, 120°40′W	4%	7	1	0	1
Point Reyes National Seashore^AF^ (Marin Co.)	Central coast California	PR	38°00′N, 122°46′W	38%	3	0	0	0
Midpeninsula Regional Open Space Preserve^CR^ (San Mateo Co.)	Central coast California	SM	37°24′N, 122°18′W	85%	31	31	26	29
Big Sur/Los Padres National Forest^RD^ (Monterey Co.)	Central coast California	LP	36°00′N, 121°27′W	34%	17	15	5	9
Total seedlings					1388	987	449	799

* Collection performed by: ^RD^the authors; ^CR^Cindy Roessler, staff, & volunteers, MROSP; ^AF^Alison Forrestel & staff; PRNS.

† Nettel et al. (2009).

‡ % germinated (from a maximum of 66 seed per family, as available).

§ Numbers of parent trees used for each experiment, determined by germination and seedling availability, except bottom row, which represents offspring.

### Nursery growth measures: N

Prior to planting, mean seed weight was recorded for each seed-parent family group; afterward, plants were visited at monthly intervals to record germination date. At the end of each of three growing seasons, stem height, basal diameter, counts of stems per individual, and the length and width of the longest leaf on each individual were recorded. Total leaf count and trichome density (the average of two counts made using an optical micrometer and dissecting microscope on each of the midvein and blade) were also recorded at year 1; trichome density has been variously associated with resistance to insect herbivory and with susceptibility to pathogens (Lake and Wade 2009; Monier and Lindow 2004). At year 2, each plant was given a score for herbivore damage ranging from 0 to 4, corresponding to the portion of damaged plant tissue (Table 2).

**Table 2:** Measures and dates of data collection for experiments N, R1, R2, and F. Date is presented as months from December 2006, when seeds were sown

Measure	Units	Months from Planting
Nursery growth (N)		
Average maternal seed weight	g	0
Germination date	Days	0–6
Stem height*	cm	9, 21, 33
Basal diameter	cm	9, 21, 33
Length longest leaf	mm	9, 21, 33
Width longest leaf*	mm	9, 21, 33
Stems	Count	9, 21, 33
Midevein trichome density*	cm^−2^	9
Leaf blade trichome density*	cm^−2^	9
Leaves*	Count	9
Herbivory	Score, 1–4	21
Resistance assays (R1)		
Lesion length in detached, inoculated leaves*	mm	14
Leaf length*	mm	14
Resistance assays (R2)		
Lesion length in detached, inoculated leaves*	mm	30
Lesion length in inoculated stems*	cm	34
Stem height*	cm	34
Field disease trial (F)		
Status	Score, 0–1	19, 24, 29, 35, 54
Stem height	cm	13, 24, 35, 54
Basal diameter	cm	13
Presence of stem lesion	Score, 0–1	19, 24, 29, 35, 54
Leaves with necrotic lesions†	Count	19, 24, 29, 35, 54
Dead leaves†	Count	19, 24, 29, 35, 54
All leaves†	Count	13, 19, 24, 29, 35, 54
Herbivory*	Score, 1–4	19, 24, 29, 35, 54
*Phytophthora ramorum* isolation‡	Score, 1–3	19, 29

* ln(*x*) transformed for analysis.

† ln(*x* + 1) transformed for analysis.

‡ 3-level factor (0 not tested, 1 negative, 2 positive) collapsed to 2 levels (0–1, 2) in final survival model (df = 2, χ^2^ = 0.086, *P* = 0.958).

### Inoculation assays: experiments R1 (February 2008) and R2 (June 2009)

*Phytophthora ramorum* isolate Pr52 (CBS110537; ATCC MYA-2436) was used for all inoculations. Pr52, isolated in 2000 from *Rhododendron* spp. in Santa Cruz, CA, is representative of one of the NA1 clonal lineages dominant in US forests (Ivors et al. 2006; Mascheretti et al. 2008) and had been reported highly pathogenic to *Umbellularia californica* leaves and *Quercus agrifolia* stems (Hüberli and Garbelotto 2011).

Mycelial-plug inoculations (described in detail in Hayden et al. 2010) were performed in experiment R1 on 2–4 detached mature leaves each from 10 to 15 seedlings per family, as available (Tables 1 and 2). Briefly, a plug of mycelia was placed on the cut petiole of each leaf, and then incubated in moist chambers, with leaves originating from a single seedling distributed across different chambers. Detached-leaf and intact-stem assays were conducted in experiment R2, on the subset of families with minimum five seedlings not assayed in R1 (Table 1). The terminal mature leaf from each seedling was removed and retained for subsequent inoculation, and each stem was inoculated at the wound site using a zoospore suspension of Pr52 (Hayden et al. 2010) (Table 2). Twenty-four seedlings were randomly selected for sterile-water-control inoculations.

### Field resistance study: experiment F

In January 2008, 800, 12-month-old seedlings (Table 1) were transplanted at 1-m intervals into 10 blocks. Each block was under the canopy of an *U. californica* with confirmed *P. ramorum* infection, in a mixed evergreen forest in the Santa Lucia Preserve, Carmel Valley, CA. Sixteen seedlings per family (except SM-18, with 15) were randomly assigned four locations in each of four blocks.

Status, growth, and disease symptoms were assessed at five different dates (Table 2). To confirm pathogen presence experiment-wide, limited diagnostic testing occurred at 7 and 17 months post-transplanting. Diagnostic tests were carried out via either polymerase chain reaction (Hayden et al. 2006), or isolation by culture (Davidson et al. 2003). Dead plants and living tissue with symptoms of *P. ramorum* infection were sampled if doing so would not destroy the stem or primary symptoms.

In late 2008 and early 2009, 147 seedlings suffered severe rabbit herbivory and were excluded from analyses. All seedlings were subsequently caged with wire mesh.

### Statistical analyses

#### Additive genetic variances: N, R1, R2

Variance for each measured trait or index from experiments N, R1, and R2 was modeled using the Mixed procedure in SAS v. 9.2 (SAS Institute, Cary, NC, USA) unless otherwise noted.

Tree size (N) was modeled using a repeated measures model with autoregressive subject variance as follows:


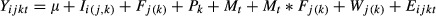
(1)

where *Y*_*ijkt*_ is the dependent variable for the random subject of the *i*th seedling (*I*) nested within the *j*th parent (*F*) and the *k*th source population (*P*), measured at the *t*th sampling time (*M*), with covariate of parental mean seed weight (*W*). μ is the trait mean, *M*_t_*F_*j(k)*_ is the time by parent interaction, and *E*_*ijkt*_ is the residual. Seedling, parent, population, and the time by parent interaction were modeled as random effects.

A similar model was used for the nonrepeated N measures:


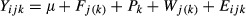
(2)

Visual inspection of variables’ distributions and models’ diagnostic plots, and tests for normality using the UNIVARIATE procedure indicated that distributions for germination date, herbivory score, and stem counts were neither normal nor log-normal. They were thus modeled as generalized linear mixed models (GLMM) using the GLIMMIX procedure. Error distributions and linear-link functions were chosen by inspecting model diagnostics and over- or under-dispersion of residuals under frequently used distributions and links for the given data type. The final models employed a negative binomial distribution for germination date, Poisson distributions for herbivory score and stem count, and a log-linear link for each model.

R1 and R2 leaf lesions grew linearly on the midrib, never reaching full leaf length. Lesion size, the inverse of quantitative resistance, was modeled:


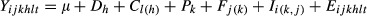
(3)

where *Y*_*ijkhlt*_ is the lesion length in the *t*th observation of the *i*th seedling of the *j*th parent from the *k*th population inoculated on the *h*th day (*D*) in the *l*th chamber (*C*). All effects except day were random. Stem lesion lengths were similarly modeled as:


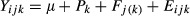
(4)

Variance components were extracted from the REML estimates of eqns 1–4. Total phenotypic variance (*V*_T_) was estimated from each equation as the sum of variance components; *V*_F_ is used here to designate the estimated maternal variance component. Preliminary data have indicated 10% selfing and 5–10 pollen parents per mother in tanoaks (R. Dodd, unpublished data); with those assumptions, *r* ≈ 0.33 (Squillace 1974). Using these parameters, additive genetic variance (*V*_A_) and narrow-sense heritability (*h*^2^) (Falconer 1989) were estimated from equations 1–4 as:


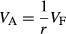
(5)


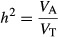
(6)

Heritability was estimated only for traits whose distributions were approximately normal or log-normal. Confidence intervals for narrow-sense heritability (*h*^2^) were constructed from the Dickerson (1969) approximation of variance of heritability, which tends to be conservative (Dieters et al. 1995):


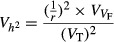
(7)

where 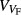 is the variance of the estimate of the maternal family variance component, computed from the square of the component's standard error, which is the linear predictor's standard deviation (SAS Institute, Inc. 2008). Confidence limits were estimated from the square root of 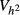 multiplied by the *Z*-statistic.

The coefficient of additive genetic variation (*CV*_A_), the additive genetic variance scaled by population mean values, is sometimes preferred to *h*^2^ as a measure of a population's potential response to selection, or evolvability (Houle 1992). On a log scale, *V*_A_ is a measure of proportional evolvability (Hansen et al. 2011). *CV*_A_ was estimated per Houle (1992):


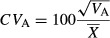
(8)

where 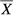 is the population mean trait value.

### Survival analysis: F

Variance in the rate of seedling deaths in response to growth and symptom data measured within the field study was likewise modeled using a GLMM. We extended the generalized linear model method of Allison (1995), which allows time-dependant covariates, to further account for unseen, random sources of covariance by using a mixed model format in the R package lme4 v. 0.999375-40 (Bates et al. 2011; R Development Core Team 2011). The probability of death at each sampling time (status 1, vs 0 if living or censored) was modeled as a function of each field-data covariate at the time previously and accounted for the expectation of a temporally increasing probability of death by a model offset of time to sampling. Censorings occurred 42 months post-transplant, when 50 seedlings were made inaccessible by fallen, SOD-symptomatic, mature tanoaks from the surrounding forest. Model terms were selected by backwards stepwise reduction to minimize AIC. Each measure of field growth or symptoms (Table 2) was tested for inclusion, culminating in the model:


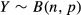
(9)


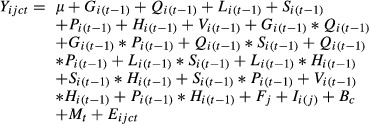
(10)

where *Y*_*ijct*_ is the binomially distributed status, with frequency of death *p*, of the *i*th seedling in the *j*th family and *c*th block (*B*) at the *t*th sampling time, occurring *M* months post-transplant*. G* is total count of leaves, *Q* is the count of dead leaves, *L* is the count of lesioned leaves, *S* is the presence of a stem lesion, *P* is a positive detection of *P. ramorum* by PCR or culture, *H* is height (cm), and *V* is herbivory score (0–4), in the *i*th seedling at the previous sampling time. All covariates were recorded in the field, within experiment F. Block, family, seedling, and sampling time were random factors. The natural-log-transformed time to sampling in months [ln(*M*_*t*_)] was offset, and a complementary log-log link, which is the linear transform for the probability of surviving beyond some time, was employed. Population of origin was omitted because its inclusion caused model convergence failures, likely because of unbalanced design.

To assess whether, despite the wide uncertainties surrounding additive genetic variance, laboratory resistance and growth experiments could help predict field survival of related individuals in the absence of other information, we modeled the likelihood of death as a function of the breeding values for growth and resistance traits estimated from the trees’ siblings in the nursery. For each of the 34 families with data from all of N, R1, and R2 experiments, deaths were summed across blocks and expressed as the proportion of deaths per family members at each sampling time. The family best linear unbiased predictors (BLUPs) for traits with approximately normal or log-normal distributions were tested for model inclusion as above, except that N stem height (correlated with leaf count and diameter) and N midvein trichome density (correlated with blade trichome density) were left out *a priori* to reduce multicollinearity and overparameterization:


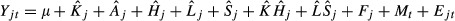
(11)

where *Y*_*jt*_ is the binomially distributed frequency of deaths *p* in the *j*th family at the *t*th sampling time, occurring *M* months post-transplant. 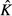 is the BLUP for leaf blade trichome density, 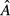 is nursery leaf length BLUP, 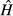 is R2 stem height BLUP, 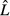 is R2 leaf lesion length BLUP, and 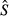 is R2 stem lesion length BLUP.

To further assess the practical impact of observed differences, we imagined our field trial as a post-disturbance restoration project and used bootstrapping to model the effect of proposed family screening criteria on survival, had they been applied before out-planting. Survival of 10 000 sets of 10 families selected randomly with replacement from those included in experiment R2 was compared with sets of 10 drawn from groups meeting a threshold of (i) R2 stem or (ii) leaf resistance (arbitrarily defined as families whose BLUP for lesion was in the lower quartile of the size range for either type), (iii) tall stems in R2 (upper quartile), or where (iv) either type of resistance was present and R2 height was moderate or better (upper 75th percentile).

## Results

### Establishment of a common garden

Of the initial 10 308 acorns from 112 seed parents, 4147 germinated and were divided into experimental populations (Table 1). Growth studies were conducted on all available seedlings. Laboratory resistance assays (R1, R2) and the field disease trial (F) required a minimum number of individuals, so each of these included only a subset of the families studied for growth (N) (Table 1). The 50 families in experiment F were a subset of those tested in N and R1; the 34 families in R2 were all included in F. Lower germination rates from some populations led to an unbalanced representation in later experiments: experiment F included 29 families from San Mateo (SM), 11 from the Klamath region (OB), nine from Monterey (LP), and one from the Sierra Nevada (BL).

### Nursery growth measures: N

After 3 years, stem heights ranged from 5 cm to 2.51 m, with 1–8 stems per individual. Leaves ranged from 3.7 to 17.2 cm in length and 1.8 to 14.6 cm in width. Mean maternal seed weight had significant effects on the nursery growth measures of height, diameter, and leaf length and width (Table 3). Additive genetic variance parameters were low, with low-to-moderate heritability, ranging from of *V*_A_ = 0.007 and *CV*_A_ = 2.8 (log-transformed mm) and *h*^2^ = 0.14 for leaf width, to *V*_A_ = 0.224, *CV*_A_ = 16.5 (log-transformed mm^−2^) and *h*^2^ = 0.38 for leaf blade trichome density (Table 4). Linear predictors for family breeding values are provided as supplementary information (Table S1).

**Table 3:** Fixed effect estimates for individual trait analyses of variance. Models were run individually for each dependent variable (Eqns 1–4); see Table 4 for random effects. No fixed effects were included in resistance assay models (Eqns 3–4). F-ratios are given for models with normal distribution; if other than normal, error distribution is specified and *t*-ratios are reported

Dependent variable (Experiment/units)	Fixed effect	DF	*F/t*	*P*
Stem height (N: ln(cm))*	Seed weight	3292	8.98	0.003
	Time	168	2891.48	<0.0001
Stem diameter (N: mm)*	Seed weight	3295	13.42	0.0003
	Time	168	950.54	<0.0001
Leaf length (N: mm)*	Seed weight	3285	10.30	0.0013
	Time	168	1003.31	<0.0001
Leaf width (N: ln(mm))*	Seed weight	3282	8.56	0.0035
	Time	168	440.36	<0.0001
Number of stems (N: ln(*x*))†	Seed weight	1023	0.81	0.416
Number of leaves (N: ln(*x*))†	Seed weight	63.6	1.69	0.199
Midvein trichome density (N: ln(cm^−2^))	Seed weight	67.2	1.85	0.178
Blade trichome density (N: ln(cm^−2^))	Seed weight	73.7	1.63	0.205
Herbivory score (N: *x*)†	Seed weight	1080	0.91	0.363
Germination date (N: days from planting)‡	Seed weight	1192	1.45	0.146

* Repeated measures GLMM with time as a fixed, classed effect.

† Poisson distribution.

‡ Negative binomial distribution.

DF, degrees of freedom.

**Table 4:** Variance component estimates for random effects in individual trait analyses of variance (Eqs 1–4). Note that variance (*V*_A_) was significantly different from 0 at *P* < 0.05 for all traits *except* R2 leaf lesion length and R2 stem lesion length. Confidence intervals for *h*^2^ were estimated with the Dickerson (1969) approximation (Eq 7)

Dependent variable	Random effect	Variance	SE	*P*	*V*_A_	*CV*_A_	*h*^2^ (95% CI)
**Stem ht (N: ln(cm))**	Population	0.034	0.024	0.082	0.038	4.08	0.15 (0.06–0.25)
	Parent (population)	0.013	0.004	0.002			
	Tree × time (population)	0.009	0.001	<0.0001			
	Residual	0.194	0.006	<0.0001			
Stem diam (N: mm)	Population	0.128	0.135	0.172	0.646	11.47	0.11 (0.01–0.21)
	Parent (population)	0.213	0.094	0.012			
	Tree × time (population)	0.383	0.070	<0.0001			
	Residual	4.948	0.145	<0.0001			
Leaf length (N: mm)	Population	13.432	11.444	0.120	82.882	17.54	0.31 (0.17–0.45)
	Parent (population)	27.351	6.318	<0.0001			
	Tree × time (population)	8.969	2.302	<0.0001			
	Residual	215.040	5.846	<0.0001			
Leaf width (N: ln(mm))	Population	0.009	0.007	0.081	0.007	2.82	0.14 (0.05–0.22)
	Parent (population)	0.002	0.001	0.001			
	Tree × time (population)	0.002	0.000	<0.0001			
	Residual	0.037	0.001	<0.0001			
Number of leaves (N: ln(*x*))	Population	0.019	0.014	<0.0001	0.027	6.60	0.19 (0.07–0.31)
	Parent (population)	0.009	0.003	0.001			
	Residual	0.115	0.005	<0.0001			
Midvein trich dens (N: ln(cm-2))	Population	0.024	0.024	0.162	0.144	12.70	0.33 (0.16–0.50)
	Parent (population)	0.047	0.013	<0.0001			
	Residual	0.363	0.015	<0.0001			
Blade trich dens (N: ln(cm^−2^))	Population	0.071	0.059	0.113	0.224	16.48	0.39 (0.21–0.56)
	Parent (population)	0.074	0.017	<0.0001			
	Residual	0.435	0.018	<0.0001			
Leaf length (R1: mm)	Population	38.564	30.466	0.103	75.062	15.70	0.32 (0.20–0.44)
	Parent (population)	24.771	4.898	<0.0001			
	Residual	169.340	4.030	<0.0001			
Leaf lesion length (R1: ln(mm))	Population	0.001	0.001	0.187	0.006	2.98	0.11 (0.00–0.21)
	Parent (population)	0.002	0.001	0.001			
	Seedling (parent, population)	0.013	0.001	<0.0001			
	Incubation box (date)	0.002	0.000	0.001			
	Date	0.000	0.001	0.204			
	Residual	0.038	0.001	<0.0001			
Stem ht (R2: ln(cm))	Population	0.012	0.016	0.2347	0.045	6.10	0.32 (0.05–0.58)
	Parent (population)	0.015	0.006	0.0102			
	Residual	0.115	0.008	<0.0001			
Leaf length (R2: mm)	Population	0.000			37.716	9.46	0.25 (0.00–0.50)
	Parent (population)	12.446	6.358	0.0251			
	Residual	138.170	9.790	<0.0001			
Leaf lesion length (R2: ln(mm))	Population	0.001	0.003	0.374	0.012	4.37	0.08 (−0.03–0.21)
	Parent (population)	0.004	0.003	0.108			
	Incubation box	0.022	0.012	0.037			
	Residual	0.118	0.008	<0.0001			
Stem lesion length (R2: ln(cm))	Population	0.000			0.052	17.07	0.10 (−0.15–0.36)
	Parent (population)	0.017	0.022	0.214			
	Residual	0.479	0.045	<0.0001			

### Inoculation assays for resistance: R1, R2

Detached-leaf assays for resistance of nursery seedlings to *P. ramorum* were significantly affected by seed parent and by incubation box, with low additive variance parameters in both R1 and R2 (Table 4). R1 leaf lesion lengths ranged from 4 to 54 mm, and R2 leaf lesions were 3–47 mm. After 5 months incubation, stem lesions in 2.5-year-old seedlings (R2) ranged from 0 to 23.5 cm (mean 3.0 cm) in inoculated trees, compared to 0–3.5 cm (mean 0.2 cm) in mock-inoculated trees. Estimates of additive genetic variation at R1 had greater precision than R2, in which the sample size was halved by necessity. In R2, only the parental variance component for R2 tree size was significantly different from zero; those for resistance measures were not (Table 4).

### Trait correlations: N, R1, R2

After sequential Bonferroni correction (Sokal and Rohlf 1995), there were statistically significant pairwise correlations among nursery growth traits, but not between resistance and growth traits, nor perhaps surprisingly, among resistance traits (Table S2). Stem height was positively correlated with its diameter, as well as leaf length, width, and number, and had a nonsignificant tendency toward moderate positive correlation with the number of stems. Trichome density measures were correlated with each other and with a longer time to germination, significantly so for blade trichome density, and tended toward negative correlation with measures of size. R1 leaf lesion sizes were significantly correlated with R1 leaf lengths. Leaf length and stem height were measured in multiple experiments and therefore at different times and under different growing conditions; these tended toward positive but somewhat weak correlations across experiments (*r* = 0.24–0.36).

### Survival analysis: F

Forty-two months after transplanting seedlings into an infested site, family survival ranged from 0 to 100% within blocks, and from 46% to 100% across blocks, after excluding 147 plants lost to herbivory, as well as two seedlings recorded alive after having been previously declared dead. *Phytophthora ramorum* was isolated in 22 of 67 sampled plants 7 months after transplanting, and in 70 of 159 plants sampled 17 months after transplanting, indicating natural inoculation in the first months of the study, with the variable infection rates and some pathogen escapes expected in a rain-splash dispersed pathogen. After exclusions, there were 2763 observations of 650 seedlings in 50 families, across 10 blocks, and five sampling dates. A GLMM survival model for seedlings indicated that the number of dead leaves was strongly predictive of the likelihood of death at the following sampling (Table 5, estimate = 3.27, *Z* = 3.09, *P* = 0.002), and the number of leaves with lesions was slightly less so (estimate = 1.10, *Z* = 2.45, *P* = 0.014). The total number of leaves was positively associated with survival (estimate = −0.68, *Z* = −3.07, *P* = 0.002), as was an herbivory score of 2 (corresponding to 25–50% of above-ground mass showing damage, estimate = −4.56, *Z* = 2.59, *P* = 0.03). There were significant interactions among symptoms on the risk of death: each of the interactions for total leaves by dead leaves, total leaves by positive finding of *P. ramorum*, and height by lesioned leaves had negative estimates; in each case, a large value for the growth measure compensated for symptoms, driving down the risk of death for larger trees. The observed interaction of stem lesion presence and *P. ramorum* detection was confounded by the sampling methodology for *P. ramorum*; because of our desire to sample with as little interference of disease progress as possible, *P. ramorum* isolation was seldom attempted from stem lesions and more frequently from leaves.

**Table 5:** Fixed effects in GLMM survival analysis of a field disease trial (F); below-zero estimates are associated with a decreased risk of death and increased life span, while values above zero indicate an increased risk of death. Two models were run: first, likelihood of an individual tree's death dependent on field data covariates (Eqn 10); second, likelihood of family-level deaths dependent on predictors (BLUPs) estimated from nursery growth and resistance experiments (Table S1, Eqn 11)

Effect	Estimate	SE	Z	*P*
Field covariate				
Number leaves total	−0.678	0.221	−3.067	0.002
Dead leaves	3.267	1.058	3.088	0.002
Lesioned leaves	1.096	0.447	2.453	0.014
Stem lesion presence	2.275	1.230	1.850	0.064
Herbivory category 1	−0.204	0.758	−0.269	0.788
Herbivory category 2	−4.458	2.058	−2.166	0.03
Herbivory category 3	−0.780	1.386	−0.562	0.574
*Phytophthora ramorum* detection	11.033	9.514	1.160	0.246
Height	−0.014	0.026	−0.523	0.601
Lesioned leaves × stem lesion	−1.880	0.879	−2.139	0.032
Lesioned leaves × height	−0.076	0.037	−2.092	0.036
Stem lesion × dead leaves	1.344	0.739	1.819	0.069
Stem lesion × detection	−6.389	3.233	−1.976	0.048
Stem lesion × height	−0.186	0.102	−1.817	0.069
Total leaves × dead leaves	−1.206	0.477	−2.531	0.011
Total leaves × detection	−16.949	11.405	−1.486	0.137
Dead leaves × detection	12.134	7.714	1.573	0.116
Herbivory 1 × height	0.004	0.061	0.058	0.954
Herbivory 2 × height	0.247	0.095	2.587	0.01
Herbivory 3 × height	0.098	0.091	1.081	0.28
Detection × height	0.623	0.303	2.058	0.04
BLUP				
N: leaf blade trichome density	−0.163	0.429	−0.381	0.703
N: leaf length	0.047	0.024	1.942	0.052
R2: height	−5.200	1.503	−3.459	0.0005
R2: leaf lesion length	−1.258	3.608	−0.349	0.727
R2: stem lesion length	9.483	2.515	3.771	0.0002
N: blade trichomes × R2: height	11.674	4.686	2.491	0.013
R2: leaf lesion × stem lesion	197.762	62.794	3.149	0.002

BLUP, best linear unbiased predictors.

A second survival model, for the risk of death at the family, rather than seedling, level was performed using linear-model predictors (Table S1) from nursery growth and resistance experiments as effects. Predictors for tall seedlings in experiment R2 were significantly associated with higher family-level field survival, while predictors for large lesions (i.e., less quantitatively resistant) were significantly associated with mortality (Table 5). There was a strong, significant interaction of R2 leaf and R2 stem lesion predictors, where smaller predictors (more resistance) by either or both measures were associated with greater survival. There was a weaker interaction of leaf blade trichome density with R2 stem height (Table 5).

**Figure 1:**
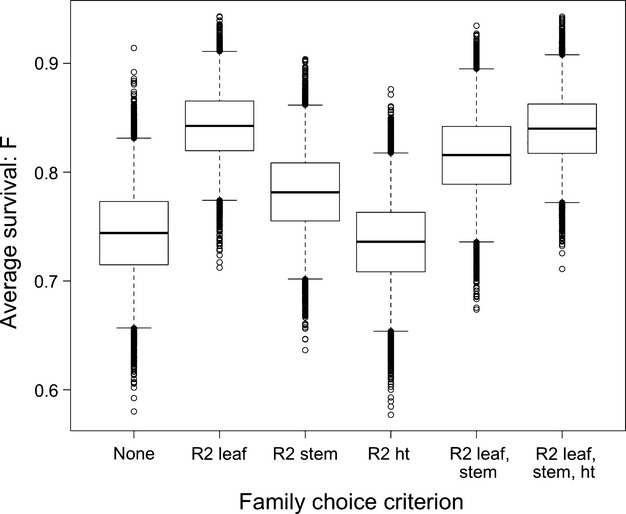
Mean field survivorship in hypothetical sets of 10 seedling families (10 000 sets, drawn with replacement), selected either from the population as a whole (None), or on the basis of disease resistance in R2 assays in leaves (R2 leaf), stems (R2stem), and/or by height (R2 ht). Boxes represent the interquartile range around the median (horizontal line), while whiskers (dashed lines) extend to 1X the interquartile range from the box, which in this case approximates the 95% bootstrap CI.

Application of the proposed BLUP criteria for nursery traits identified 8–10 families by each. Average survivorship was highest in families identified as either R2 leaf resistant or R2 leaf or stem resistant together with moderate tall height (Fig. 1). Survivorship of those chosen for stem resistance was also greater than for no selection, but less so, while those chosen for height alone performed no better than the population average. All 95% bootstrap confidence intervals overlapped.

## Discussion

We studied growth and sudden oak death-resistance traits in open-pollinated tanoak seed families in concurrent nursery and field experiments. Under field disease conditions, strong growth and signs of disease acted in opposition in predicting time of death; likelihood of survival with signs of infection were increased with trees’ strong growth. Despite little additive genetic variation for disease resistance in nursery studies, we found similar interactions when using breeding values for nursery growth and resistance traits to explain survival of their field-planted siblings, with stem height and resistance in 2nd year stem assays indicators of good field performance. In a field setting with interval sampling, causes of death are not known with certainty. Even with a positive finding of the pathogen, it only rarely possible to distinguish a seedling killed by disease from one which tolerated infection, but died of some other cause. Conversely, the lack of finding of *P. ramorum* in a dead seedling does not rule out the pathogen as the cause of death; oomycetes are difficult to isolate from dead, woody tissue, even with sensitive diagnostic methods (Hayden et al. 2004). We do not presume that the observed deaths were caused only by sudden oak death, but by a number of factors. These factors can reasonably be assumed to include infection by *P. ramorum*, among others, and may possibly include unmeasured, correlated characters. The results of two survival analyses highlight the interactions of disease and other physiological processes in determining field survival.

A survival analysis modeling the risk of seedlings’ death in an infested field site using field-recorded data revealed contrasting effects of growth and disease phenotypes on seedlings’ field survival time. The development of disease symptoms tended to predict seedlings’ death, but death could be held off in the presence of symptoms by strong growth, at least within the study's time period. Field herbivory in the low-intermediate level was associated with increased survival. It is possible that herbivores preferred more vigorous plants, or that herbivory directly contributed to survival by stimulating growth. Conversely, herbivores may have removed diseased tissue or reduced the surface area of infection-prone newly emerged leaves during times of active sporulation. There was an interesting, significant interaction of lesioned leaves and stem lesion presence, such that in seedlings with stem lesions, the number of lesioned leaves was positively associated with survival, while there was a marginally significant trend in the opposite direction for the count of dead leaves by stem lesion presence (Table 2). The response surface is so complex as to make inference difficult, but we speculate that the presence of living, lesioned leaves in infected trees may have indicated some degree of resistance; trees that were able to restrict lesion spread in stems may have also been able to restrict lesion spread within their leaves, holding off leaf death. Alternatively, leaf lesions may have indicated an earlier stage of disease, although the presence of stem lesions would seem to preclude this explanation (anecdotally, leaf deaths tend to predate stem lesions, even at this very small size class). Further monitoring of the time to death of seedlings with leaf lesions will distinguish between these possibilities, or whether the marginally significant interaction was an artifact of correlations among measured traits or with some unmeasured variable.

In seedlings that remained in a nursery, the low-to-mid-level narrow-sense heritability, we observed for growth traits was in line with other forest trees (Cornelius 1994; Cappa et al. 2010; Scotti et al. 2010), as was the strong correlation we observed among stem and leaf growth (Table S2) (Marron et al. 2007). We observed lower heritability in resistance traits than growth traits, indicating a greater relative contribution of environmental effects, including considerable among incubation box differences; in the 2nd year of testing, heritability was not significantly different from zero. Water dynamics, endophytes, and/or nutrients may have varied among plants, despite the common-garden setting, in addition to any variation among inoculum plugs or zoospore suspensions; each may play a role in disease resistance (reviewed in Agrios 2005). Estimates for *CV*_A_ or log-transformed *V*_A_ are a better approximation of evolvability than *h*^2^ (Hansen et al. 2011); these, too, were low, and in the case of R2 resistance traits, statistically insignificant.

Despite the uncertainty surrounding estimates for their additive genetic variance, there were strong and significant correlations between seed-parent family predictors (BLUPs) in nursery experiments and family average survival in the field. Family survival was directly, positively correlated with BLUPs for each of height and reduced stem lesions in the R2 experiments and strongly correlated with predictors for reduced lesions in both stems and leaves together. The nonsignificance of estimates for *V*_A_ in the R2 resistance experiments makes interpretation of these findings difficult, however. While genetically based disease resistance might have been in some small or large part responsible for variation in survival of tanoak families, the observed effects may have also been due to confounding environmental factors or correlated traits that simultaneously increased apparent disease resistance and field survivorship. Such unseen correlations may explain the significant interaction of N trichome density with R2 height, which otherwise eludes simple interpretation.

At a landscape scale, nursery and field disease garden studies should be replicated throughout the host range to disentangle adaptation to local conditions from other traits affecting fitness. The unbalanced representation of populations in the field disease garden, and in particular the R2 resistance experiment, complicated testing population-level differences in traits and field survival. There were families from each of close populations (SM and LP) and distant (OB) among the four with highest average survival. However, none of these OB families had sufficient germination for seedlings to be included in R2 assays, so it is impossible to know whether their high field survival could have been predicted with nursery assays.

Loblolly pines have shown a pattern in more highly heritable resistance to fusiform rust similar to that we observed, whereby nursery assays identified field-resistant families, but not all field-resistant families were resistant in the nursery (Powers and Kuhlman 1987; Isik et al. 2008). Several factors may explain the directional discrepancy. First, under natural disease dynamics, infection rates are not expected to be uniform, and many of the tanoak seedlings in our study may have escaped early infection. Furthermore, a single *P. ramorum* isolate and high concentrations of inoculum were used for laboratory assays, so host-genotype interactions or inoculum threshold effects could explain high fitness of some seemingly susceptible families. The *P. ramorum* population in the area of the field study has been reported to have relatively low genetic diversity relative to other California populations (Mascheretti et al. 2009), and no relationship between microsatellite multilocus genotypes and *P. ramorum* virulence within the major North American lineage has been reported (Hüberli and Garbelotto 2011; Kasuga et al. 2012). Quantitative differences in virulence have been reported among three major lineages of *P. ramorum* worldwide (Elliott et al. 2011), and to differ with the host species from which it was isolated (Hüberli and Garbelotto 2011; Kasuga et al. 2012). However, Kasuga et al. (2012) found that virulence phenotypes were not associated with multilocus genotype within the major North American lineage but were in fact depending on host infection history, with a loss of virulence after infection *Quercus* spp. Nonetheless, *P. ramorum* isolates’ pathogenicity tends to be stable across hosts in inoculation studies (Hüberli and Garbelotto 2011). Still, as-yet-unobserved strain-specific resistance may be important in natural infection dynamics, and studies should be extended to characterize the durability of nursery resistance across pathogen genotypes and geographic locale.

Nursery studies may not have included all of the factors contributing to fitness, including some forms of disease resistance; conversely, observed disease resistance may be the result of correlation with other traits or environmental effects, as evidenced by low additive genetic variation. Each of the assays assessed different qualities and should be considered a different character. Detached-leaf assays challenged wounded tissue with vigorously growing mycelia, so only the ability to resist spread after initial infection was tested. The response was very different at 13 months (R1) and 30 months (R2), probably as a result of nursery environment, diminishing maternal effects, or developmental differences. Disease resistance responses can differ between young seedling and adult stages (reviewed in Burdon 1987); R1 leaf resistance, while differing significantly among families, had no value as a screen for post-transplant fitness. Because they employed zoospores, rather than already-growing mycelial plugs, the stem assays were comprised of two components: qualitative susceptibility to initial infection and the quantitative degree of lesion spread in infected individuals. Each of these components may have genetic and environmental contributions. Gene-for-gene host–pathogen interactions are usually characterized by qualitative responses, with some exceptions (Bent and Mackey 2007); these interactions are thought to be more easily overcome by pathogen evolution, while quantitative resistance—in the classic case, underlain by several to many host genes—is believed to be more durable to pathogen variation (Carson and Carson 1989; Kinloch et al. 2008).

To further assess the potential utility of nursery resistance screening assays, we imagined our field trial as a restoration project. Screening with our proposed 2nd year leaf resistance criterion alone resulted in an increase in survival of 10 percentage points in bootstrapped replicates. If these results are broadly applicable, restoration projects that use seeds from parents identified here or by future screenings could see more trees surviving in the field after only 5 years. Detached-leaf assays combined with continued monitoring and validation should be considered as an addition to programs planting tanoaks for ecological restoration. Because the seedling remains intact and uninfected after screening, these assays can identify preferred individuals within families before transplanting, increasing the value of this weakly heritable trait. Combined stem and leaf assays can identify more families than can either assay alone, but stem assays are more expensive, requiring controlled growth chambers and the sacrifice of tested seedlings. If the trend toward higher proportionally scaled evolvability of stem resistance traits over leaf resistance is confirmed in future research, however, a greater long-term gain is to be expected by selection for stem traits. Long-term monitoring of the field disease garden is called for to allow further disease progression. In the relatively short term, further monitoring will allow estimates of additive genetic variation to be refined, while longer-term monitoring can confirm whether higher survival associated with resistance assays are due to genetic disease resistance or some correlated trait and determine whether protective effects seen in seedlings last through maturity, whether they withstand multiple infection events, and which effects may not be observable in short-term studies (Laine 2011).

Whether or not seedling resistance to sudden oak death is correlated with adult survival in tanoaks, the differences we observed are likely to play a role in the evolutionary ecology of the forest systems in which they are endemic. First, surviving the seedling stage is an obvious, necessary precursor to adult survival and reproduction. Second, stage-structure models have found the rates of survival and *P. ramorum* infection and transmission in the smallest size classes of tanoaks to help drive the rate with which neighboring large, overstory tanoaks are extirpated (Cobb et al. 2012). Studies of SOD dynamics in mature tanoaks have demonstrated that large size-classes tend to die most quickly (Cobb et al. 2010; McPherson et al. 2010), and earlier studies showing the persistence of tanoak sprouts even under heavy disease pressure have suggested environmental and stochastic effects as the cause (Cobb et al. 2010; Ramage et al. 2011). Our work indicates that resistance effects are likely to also play an important role in determining survival, just as variation in resistance within *Q. agrifolia* and *U. californica* may play a role in disease (Anacker et al. 2008; Dodd et al. 2008; Hüberli et al. 2011). Even if the relationships we observed between laboratory-observed resistance and field survival rates turn out to be limited to seedling and sapling stages, current models (Cobb et al. 2012) suggest that these may have an important, immediate ecological impact. The differences we report suggest that models of disease dynamics may be considerably refined by using empirically determined resistance parameters.

The challenge is now to understand how seedling survival correlates to adult survival and reproduction, beyond the obvious requirement that trees survive the seedling stage to reach reproductive age, to refine estimates of variance and heritability, and to understand the role of pathogen variation in the disease interaction. The establishment of study populations and the understanding of seedling disease resistance dynamics described here represent the necessary first steps in a process that will allow us to improve predictions of the long-term outcome of this disease on tanoak. We identified families and traits with greater survivorship in a diseased environment, which may now be considered as a restoration seed source, and screening processes, which managers may use prior to planting. Together, these present tools that may be used now and provide a foundation for future developments for the research-based management of noncommercial, but ecologically dominant forest trees.

## Data archiving statement

The data reported here are archived and publicly available from the Center for Forest Provenance Data (http://cenforgen.forestry.oregonstate.edu/).

## Supporting Information

**Table S1.** Relative fitness in a field disease trial and predictors for seed parents' predicted breeding values for traits measured in nursery studies.

**Table S2.** Pairwise genetic correlations of growth and resistance character BLUPs by experiment-N, R1, and R2.
