# Supplementary material for: Scaling up from greenhouse resistance to fitness in the field for a host of an emerging forest disease
Source: Evol Appl. 2013 Jul 19;6(6):970–82. doi: 10.1111/eva.12080 (PMC3779097; doi:10.1111/eva.12080)
Supplement: Supplementary file 1 [file eva0006-0970-SD1.pdf]

Table S2 Pairwise genetic correlations of growth and resistance character BLUPs by experiment- N, R1, and R2. See Table 2 for explanation of traits and units. Correlations statistically significant after sequential correction for multiple comparisons (Sokal and Rohlf 1995) are indicated by bold type (for $\alpha = 0.05$ and 120 comparisons, critical $p = 0.0004$).

		Nursery Growth										R1		R2			
		Germ	St ht	Diam	Lf len	Lf wd	Mv tri	Bl tri	Leaves	Stems	Herbiv	Lf len	Lf les	St ht	Lf len	Lf les	St les
N	Germination date	1															
	Stem height	-0.11	1														
	Diameter	-0.10	**0.79**	1													
	Leaf length	-0.06	**0.68**	**0.51**	1												
	Leaf width	-0.19	**0.58**	**0.40**	**0.82**	1											
	Midvein trich	0.27	-0.09	-0.03	-0.20	**-0.37**	1										
	Blade trich	**0.37**	0.00	0.04	-0.09	-0.27	**0.69**	1									
	No. leaves	-0.21	**0.40**	0.34	0.16	0.13	0.02	0.00	1								
	No. stems	-0.24	0.26	0.14	0.13	0.16	-0.09	-0.24	0.13	1							
	Herbivory score	-0.02	-0.10	-0.21	-0.10	0.03	-0.16	-0.06	0.06	0.14	1						
R1	Leaf length	-0.08	0.21	0.06	0.36	0.40	-0.08	-0.14	-0.09	0.12	-0.01	1					
	Leaf lesion	-0.16	0.03	-0.09	0.17	0.18	-0.05	-0.14	0.14	0.04	0.16	**0.57**	1				
R2	Stem height	0.09	0.30	0.07	0.16	0.20	-0.02	-0.05	0.03	-0.28	0.12	0.20	0.23	1			
	Leaf length	-0.12	0.21	0.01	0.24	0.08	-0.14	-0.09	-0.05	0.11	-0.24	0.28	0.17	0.29	1		
	Leaf lesion	0.04	-0.11	-0.19	0.06	-0.05	-0.03	0.04	0.07	-0.01	-0.09	-0.11	-0.05	-0.32	0.25	1	
	Stem lesion	0.16	-0.22	-0.25	-0.30	-0.39	0.08	0.14	0.05	-0.17	0.07	-0.34	-0.33	0.18	0.11	0.19	1
