# Supplementary material for: Scaling up from greenhouse resistance to fitness in the field for a host of an emerging forest disease
Source: Evol Appl. 2013 Jul 19;6(6):970–82. doi: 10.1111/eva.12080 (PMC3779097; doi:10.1111/eva.12080)
Supplement: Supplementary file 2 [file eva0006-0970-SD2.pdf]

Table S2 Relative fitness in a field disease trial and predictors for seed parents' predicted breeding values for traits measured in nursery studies. Parent is the wild maternal parent within the population (Pop). Relative fitness is the averaged field survivorship of open-pollinated offspring divided relative to overall block survivorship, 42 months after transplanting to an infested field site. Breeding values are best linear unbiased predictors (BLUPs) from linear models (Eqs 1-4), presented a deviance from the population mean of the transformed trait within experiment (Table 2).

Pop	Parent	Rel fit F	Stem height N: ln(cm)	Diam N: mm	Leaf width N: mm	Leaf length N: mm	Nm stems N: ln(x)	Herbiv N: score	Germ date N: days	Nm leaves N: ln(x)	Midvn trich N: cm^{-2}	Blade trich N: cm^{-2}	Leaf length R1: mm	Leaf lesion R1: ln(mm)	Leaf length R2: mm	Leaf lesion R2: ln(mm)	Stem ht R2: ln(cm)	Stem lesion R2: cm
BL	BL-2		-0.063	-0.138	-0.002	-2.206	-0.015	-0.018	0.098	0.015	0.083	0.079						
BL	BL-21		-0.040	-0.174	-0.022	-3.277	-0.014	-0.017	0.007	0.016	-0.008	0.051						
BL	BL-24	0.604	0.028	-0.025	0.023	1.147	0.018	-0.130	0.037	-0.048	-0.224	-0.144	0.312	0.014				
BL	BL-4		-0.007	-0.009	-0.020	-1.678	-0.015	0.000	0.003	-0.001	0.046	0.120						
BL	BL-5		0.031	0.033	0.021	1.243	-0.015	0.054	0.001	-0.003	0.041	0.066						
BL	BL-8		0.000	0.000	0.000	0.000	0.000	0.000	0.059	0.000	0.000	0.000						
BL	BL-9		-0.007	-0.135	-0.039	1.046	-0.054	-0.001	-0.089	-0.031	0.078	-0.024						
LP	LP-10	0.830	-0.077	-0.305	-0.029	-1.419	-0.046	-0.050	-0.097	0.060	0.232	0.043	-2.287	0.051				
LP	LP-11		0.067	0.089	0.026	3.292	-0.038	-0.065	-0.033	0.081	-0.126	-0.072	-5.155	-0.015				
LP	LP-12		0.093	0.231	0.026	3.639	0.040	0.089	0.104	0.019	-0.278	-0.125	0.557	0.038				
LP	LP-13	1.025	0.074	0.091	0.008	2.514	-0.091	-0.082	-0.166	0.024	-0.057	0.277	2.280	0.032	4.522	-0.005	0.077	-0.012
LP	LP-14		-0.117	-0.480	-0.024	-3.834	-0.029	0.236	-0.123	-0.010	-0.169	0.007						
LP	LP-15		0.196	0.550	0.070	8.418	-0.011	0.140	0.116	0.084	0.132	0.087	9.012	0.030				
LP	LP-16		0.007	-0.052	-0.022	-1.027	0.061	-0.020	0.054	-0.001	0.014	0.104	1.700	-0.002				
LP	LP-19	1.332	0.015	-0.107	0.036	2.962	-0.017	0.026	-0.116	0.004	-0.164	-0.032	3.966	0.030				
LP	LP-20		0.063	0.195	0.031	4.106	-0.030	0.032	0.066	0.006	-0.093	-0.091	-0.179	-0.031				
LP	LP-22	1.301	0.004	-0.038	-0.042	-4.941	0.003	0.046	0.065	-0.080	0.198	-0.047	-0.260	-0.037	2.796	-0.048	0.169	-0.010
LP	LP-23	0.783	-0.146	-0.392	-0.036	-5.243	-0.044	-0.140	0.169	-0.045	0.041	0.263	0.090	-0.051	-0.936	0.007	-0.040	0.074
LP	LP-24	1.076	-0.062	-0.070	0.004	-3.153	0.017	-0.001	-0.027	-0.091	-0.300	-0.084	-4.728	-0.068				
LP	LP-25	1.117	0.044	0.368	0.017	3.171	0.173	-0.095	-0.140	-0.105	0.283	-0.054	3.296	-0.021	-1.693	0.031	-0.089	-0.060
LP	LP-26	1.169	-0.119	-0.204	-0.027	-2.798	0.017	-0.099	0.090	-0.004	0.083	-0.180	-7.135	-0.054				
LP	LP-27	0.994	0.019	0.305	-0.021	-1.565	0.095	-0.292	-0.243	0.059	0.105	-0.107	1.888	0.041	-1.284	-0.034	-0.143	-0.134
LP	LP-3		0.015	-0.040	0.007	-0.563	-0.015	-0.019	-0.084	0.007	-0.063	-0.096						
LP	LP-7		-0.028	-0.074	-0.023	-1.717	-0.050	0.056	0.105	0.069	0.012	-0.212	-4.877	0.013				
MD	MD-26		0.107	0.430	0.012	4.386	-0.013	-0.055	-0.068	0.057	0.091	0.024						
MD	MD-27		0.028	-0.078	-0.017	-0.097	-0.035	-0.013	0.083	0.011	0.035	0.204	-7.410	-0.037				

MD	MD-28		-0.108	-0.369	-0.004	-2.621	-0.050	0.069	0.036	-0.067	0.048	0.099	-0.988	0.019				
MD	MD-30		-0.037	-0.085	0.013	-0.375	-0.036	0.035	-0.011	-0.038	0.083	-0.040	3.974	0.006				
MD	MD-31		-0.042	-0.049	-0.003	-0.438	0.000	-0.017	0.009	0.012	-0.035	-0.091						
OB	OB-1	1.058	-0.107	-0.038	0.023	-1.620	-0.086	0.039	-0.042	-0.111	-0.281	-0.264	1.579	0.047				
OB	OB-10		-0.036	0.076	-0.026	0.611	-0.057	-0.022	0.180	0.013	0.089	0.180	0.377	0.022				
OB	OB-11	0.945	-0.009	-0.154	0.013	1.962	-0.014	-0.035	-0.158	-0.048	0.054	0.126	3.642	-0.005				
OB	OB-12		-0.002	0.064	-0.042	-5.754	0.106	0.133	-0.062	0.087	-0.211	-0.091	0.205	0.029				
OB	OB-13	1.061	-0.007	0.076	0.033	-2.024	0.013	0.026	-0.053	-0.008	-0.137	-0.042	-1.053	-0.024				
OB	OB-14		-0.025	-0.109	-0.011	-3.127	0.018	-0.081	0.004	-0.063	-0.013	-0.247	6.875	0.084				
OB	OB-15		0.106	0.116	-0.018	-0.022	0.004	-0.048	0.002	0.013	0.205	0.016	10.401	0.025				
OB	OB-16		-0.091	-0.308	0.014	-1.950	-0.050	-0.094	-0.022	-0.069	0.167	0.145	-0.639	0.011				
OB	OB-17	0.731	-0.001	-0.011	0.022	2.331	0.119	0.060	0.214	-0.066	-0.101	-0.290	-1.381	0.047				
OB	OB-18	0.818	0.001	0.212	0.018	0.251	-0.027	-0.166	-0.118	0.122	0.075	-0.091	-5.113	-0.041	4.128	0.011	0.013	0.015
OB	OB-19	0.766	0.038	-0.097	0.031	-1.227	0.124	0.064	-0.060	-0.012	0.187	0.370	4.974	0.023				
OB	OB-2	1.214	-0.027	-0.244	0.032	2.055	-0.024	0.250	-0.136	-0.072	-0.301	-0.300	1.340	-0.012				
OB	OB-20	0.768	-0.038	-0.293	-0.013	-1.235	0.027	0.000	0.038	-0.092	-0.059	0.067	-0.693	-0.082	-1.812	-0.029	-0.068	0.025
OB	OB-21	1.029	-0.044	-0.053	-0.011	-3.241	0.001	-0.053	0.116	0.074	-0.115	0.018	3.538	0.066				
OB	OB-22	0.808	0.152	0.273	0.029	6.207	0.035	-0.173	-0.022	0.095	0.125	0.391	-4.820	-0.009	-0.849	-0.013	-0.045	0.064
OB	OB-23		0.008	0.128	0.011	3.782	-0.069	-0.122	0.191	-0.067	-0.304	-0.356	2.013	-0.044				
OB	OB-24		-0.042	-0.191	0.007	1.535	0.034	-0.003	0.185	-0.060	-0.061	-0.050	-1.611	-0.077				
OB	OB-25		0.099	0.450	0.019	4.777	-0.029	-0.199	0.168	0.017	0.064	0.203	-0.373	-0.013				
OB	OB-3		-0.014	0.154	-0.043	-6.002	0.014	-0.026	-0.095	0.067	0.148	-0.039	-7.121	-0.027				
OB	OB-4		0.058	0.099	0.009	-0.680	-0.014	-0.017	0.094	-0.006	-0.009	-0.067						
OB	OB-5		-0.045	-0.126	-0.023	-1.512	-0.022	0.057	0.065	0.015	0.320	0.251						
OB	OB-7	1.335	-0.052	-0.131	-0.047	-3.187	-0.019	-0.143	0.128	0.052	0.086	0.057	-6.130	-0.035				
OB	OB-8		-0.033	-0.065	0.000	2.007	0.076	-0.092	-0.110	-0.055	-0.040	-0.302						
OB	OB-9		0.089	0.135	-0.022	1.777	0.033	0.323	-0.053	0.102	0.148	0.343	-5.615	-0.016				
PR	PR-1		-0.023	-0.434	-0.042	-6.144	-0.050	0.154	-0.011	-0.041	-0.058	-0.017						
PR	PR-12		-0.015	0.067	0.013	-1.244	-0.053	-0.101	-0.084	0.106	0.183	0.119						
PR	PR-2		0.016	0.101	0.028	1.845	-0.055	0.046	-0.005	-0.025	0.143	0.129						
SM	SM-18	1.052	0.056	0.094	-0.020	1.103	0.198	0.107	0.249	0.065	0.200	0.361	-4.304	-0.039				
SM	SM-19	0.787	0.020	0.319	-0.014	0.578	-0.005	-0.149	-0.113	0.059	0.013	0.153	-6.272	-0.040	2.807	0.057	-0.065	0.032
SM	SM-20	0.806	0.091	0.177	0.052	4.690	0.025	0.011	-0.120	0.113	-0.095	-0.173	-2.170	-0.023	-1.849	-0.007	-0.021	0.006
SM	SM-21	0.996	0.069	0.068	0.055	1.986	0.053	0.116	-0.164	0.068	-0.562	-0.716	-1.910	-0.020	-1.735	0.030	-0.079	-0.036
SM	SM-22		0.042	0.098	0.020	1.986	0.077	-0.071	-0.161	-0.055	0.054	-0.146	-4.273	-0.062				
SM	SM-23	0.950	-0.023	-0.067	-0.001	4.914	-0.105	-0.156	0.149	0.064	0.210	0.390	-0.162	-0.001	-2.889	0.067	-0.159	-0.010

SM	SM-24	0.626	0.087	0.201	0.004	3.345	0.186	0.148	-0.133	0.015	-0.137	-0.021	6.130	0.127	5.945	0.029	-0.021	0.004
SM	SM-25	1.185	0.125	0.488	0.069	6.762	0.062	-0.062	-0.236	0.101	-0.264	-0.468	4.555	0.012				
SM	SM-26	1.144	-0.105	-0.318	0.004	-0.845	0.005	0.406	-0.118	0.112	0.033	-0.135	5.204	0.116	-1.898	0.004	-0.036	-0.080
SM	SM-27	0.487	0.028	-0.171	0.004	-1.370	0.085	-0.059	-0.320	0.033	-0.122	-0.093	-3.241	-0.001				
SM	SM-28	0.621	0.024	0.084	-0.007	2.731	-0.121	-0.093	0.317	-0.003	0.156	0.298	2.428	-0.005	1.344	0.047	0.125	0.076
SM	SM-29	1.180	0.064	-0.054	0.032	3.034	0.000	0.050	0.031	0.008	0.097	0.114	6.856	-0.019	-0.164	-0.005	0.092	-0.022
SM	SM-30	1.337	-0.151	-0.654	-0.038	-5.838	-0.072	-0.102	0.138	-0.082	0.069	0.082	2.397	0.074	-1.735	-0.037	0.013	-0.061
SM	SM-31	1.196	0.136	0.112	0.056	12.191	-0.024	-0.066	-0.006	-0.015	-0.081	-0.240	1.573	0.089	1.708	-0.011	0.124	-0.070
SM	SM-50	1.200	0.072	0.569	0.030	0.245	-0.091	0.055	-0.131	0.074	-0.249	-0.103	1.571	0.024	-2.616	-0.054	0.124	-0.024
SM	SM-51	1.113	-0.019	-0.251	-0.031	-5.091	-0.051	-0.178	-0.029	0.020	0.181	0.126	4.163	0.008	2.898	-0.002	0.132	0.048
SM	SM-52	1.283	0.086	0.756	-0.038	-2.174	0.038	-0.042	-0.218	0.015	-0.077	-0.078	-4.991	-0.101	-1.103	-0.050	-0.009	0.070
SM	SM-53	0.764	0.011	0.101	-0.001	-1.072	-0.028	-0.050	0.226	-0.091	0.099	0.120	0.485	0.015	0.594	-0.013	-0.044	-0.022
SM	SM-54	1.213	-0.087	-0.486	-0.026	-5.381	0.014	0.115	-0.221	-0.049	-0.048	-0.242	-6.847	0.005	-0.520	0.049	0.054	0.043
SM	SM-55	1.009	-0.043	-0.271	-0.025	-2.643	-0.050	0.104	0.027	0.034	-0.238	-0.346	-5.414	-0.021	1.232	0.031	0.082	0.083
SM	SM-56	1.105	-0.144	-0.197	-0.081	-6.604	-0.113	0.091	0.146	-0.087	0.112	0.444	-7.731	-0.060	-1.416	0.056	-0.143	0.057
SM	SM-57	1.114	0.017	0.369	-0.035	-5.932	0.001	-0.054	0.068	-0.018	0.089	0.293	-2.795	-0.048	-0.505	0.044	-0.081	-0.068
SM	SM-58	1.073	0.055	0.402	0.033	0.396	0.006	0.069	0.107	0.014	-0.002	0.116	0.220	-0.013	-3.903	-0.039	-0.022	-0.129
SM	SM-59	1.073	0.031	-0.018	-0.005	-1.471	-0.049	-0.011	0.268	-0.121	0.175	0.191	0.071	-0.001	0.539	0.013	0.076	0.035
SM	SM-60		-0.027	-0.156	0.027	3.722	0.064	-0.002	0.034	-0.046	-0.021	-0.019	3.505	-0.025				
SM	SM-62	0.917	-0.188	-0.465	-0.112	-14.855	-0.015	0.011	-0.123	-0.022	0.141	-0.226	-0.413	0.023	-2.418	-0.016	-0.089	0.097
SM	SM-63	0.941	-0.148	-0.411	-0.002	-0.739	-0.004	0.159	0.058	-0.155	-0.002	0.067	-2.817	-0.067	0.988	-0.021	-0.132	-0.028
SM	SM-64	0.904	0.011	0.250	-0.024	0.261	-0.051	-0.050	0.277	-0.058	0.041	0.097	0.584	-0.019	0.862	-0.043	0.043	-0.022
SM	SM-65	1.100	0.020	0.460	0.031	7.535	-0.103	0.046	-0.206	-0.072	0.061	0.280	2.151	0.071	-1.840	-0.045	0.125	-0.013
SM	SM-73	1.122	-0.022	-0.132	0.018	-1.008	0.119	0.343	0.146	0.124	0.111	0.045	0.223	0.021	-2.986	-0.034	0.068	0.094
SM	SM-74	1.128	0.012	-0.066	0.057	10.403	0.107	-0.133	-0.199	-0.010	-0.336	-0.454	16.771	0.057	5.351	0.034	-0.029	-0.022
